# Biocompatibility of Collagen Membranes Assessed by Culturing Human J111 Macrophage Cells

**DOI:** 10.3390/ma2030945

**Published:** 2009-08-18

**Authors:** Claudia Gaetana Aruta, Maria Antonietta Croce, Daniela Quaglino, Deanna Guerra, Roberta Tiozzo

**Affiliations:** 1 Department of Biomedical Sciences, Section of Biochemistry, University of Modena and Reggio Emilia, Via G. Campi 287, 41100 Modena, Italy; E-Mail: claudiagaetana.aruta@unimore.it (C.G.A.); 2 Department of Biomedical Sciences, Section of General Pathology, University of Modena and Reggio Emilia, Via G. Campi 287, 41100 Modena, Italy; E-Mails: mariaantonietta.croce@unimore.it (M.A.C.); daniela.quaglino@unimore.it (D.Q.); deanna.guerra@unimore.it (D.G)

**Keywords:** collagen membrane, cell culture, proliferation, cytokine production

## Abstract

We have carried out an *in vitro* study on the interactions of human macrophages (J111 cell line) with different scaffolds made of type I and II collagen, isolated from horse tendon and from horse articular and trachea cartilage, in order to assess growth properties and biocompatibility of these membranes. We have therefore evaluated cell adhesion and proliferation as well as cytokine production considered an indicator of macrophage activation. The inflammatory response is in fact one of the major causes of collagen destruction thus interfering with cell and tissue behaviour. Moreover, the morphology of cells, seeded on membranes selected for the best characteristics, was described. Results might be relevant for *in vivo* application such ad “tissue engineering” and/or specialized cells implants.

## 1. Introduction

Collagen is the most abundant protein within the extracellular matrix (ECM). The primary structural collagen in mammalian tissues is type I, but more than 29 distinct types have been identified and exist in naturally ECM, albeit in much lower quantities. The relative concentrations and orientation of these collagens as well as their physico-chemical characteristics provide an ideal environment for cell growth both *in vivo* and *in vitro* [1]. Type I collagen has been well characterized and is ubiquitous across the animal kingdom [2]. Its excellent biocompatibility and safety, due to biological characteristics, such as biodegradability and weak antigenicity, made this matrix constituent a primary resource in biomedical applications. For this reason, allogenic and xenogenic collagens have been long recognized as one of the most useful biomaterials, that can be prepared in a number of different forms including shields, strips, sheets, sponges and beads. Many bio-medical applications have been reported: sheets as a drug delivery system for the treatment of infected tissues, sponges for burns/wounds, mini-pellet for protein delivery, basic matrices for cell culture systems [3,4]. Scaffolds of type I collagen are excellent matrix substrates providing adhesive properties for cells proliferation, migration and differentiation [5,6,7,8]. In addition, they are capable to function as a guide for cells migrating into repair areas and synthesizing extracellular matrix components assuring adequate tensile strength. Fuss *et al*. [7], for instance, used collageneous membranes for tissue “engineering” and tried to create a resistant and stable cell-matrix biocomposite with viable and biosynthetically active human chondrocytes, osteoblasts or fibroblasts. Furthermore, in different pathological conditions, collagen-based materials seem to be the most promising tool to be used as dermal or bone substitute, or as a fundamental constituent of artificial blood vessels and valves [9,10,11,12,13].

Recently, experiments have been performed showing the great potentiality of collagen for the treatment for large chondral defects in the knee and the ankle, and it has been suggested that matrix-membrane-induced autologous chondrocyte implants (MACI) [14,15,16,17,18,19] can be adopted for treating chondral and osteochondral lesions, since harvested chondrocytes can be expanded and seeded onto type I/III collagen membranes.

We have carried out an *in vitro* study with different membranes made of collagens type I and II isolated from horse tendons or articular and trachea cartilage, respectively. We have evaluated the interactions of these matrix substrates with macrophages cultured *in vitro*. The rationale to use macrophages is because these cells are involved in inflammation, immunity and repair due to their ability to phagocyte particles and cell debris, to process and expose antigenic moieties and to secrete an array of mediators such as growth factors and cytokines capable to induce and sustain the inflammatory response by attracting and stimulating other cells, but also contributing to tissue injury and fibrosis, if not adequately regulated.

In the present study we describe the behavior and the morphology of the J111 human monocyte/macrophage leukemia cell line, seeded on different collagen membranes. Cell proliferation and viability have been evaluated by structural and functional assays, whereas membranes biocompatibility has been investigated by detecting the production of cytokines as a marker of cell activation and reactivity. Data might be relevant for *in vivo* applications such as “tissue engineering” and/or for specialized cell implants.

## 2. Results and Discussion

### 2.1. Confocal microscopy

**Figure 1:**
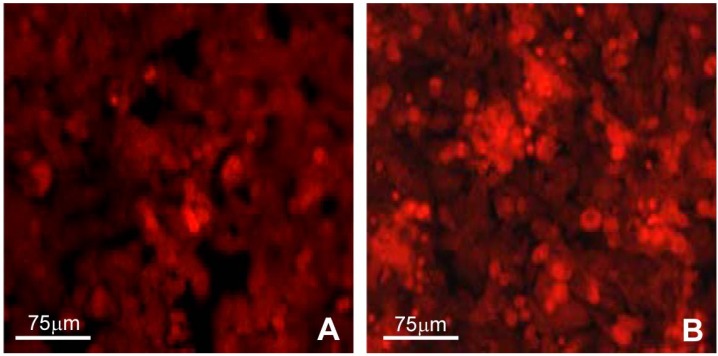
Confocal microscopy of J111 cells grown on a plastic surface for 3 (A) and 5 (B) days. Cells were labeled with propidium iodide prior to microscopy observation.

**Figure 2:**
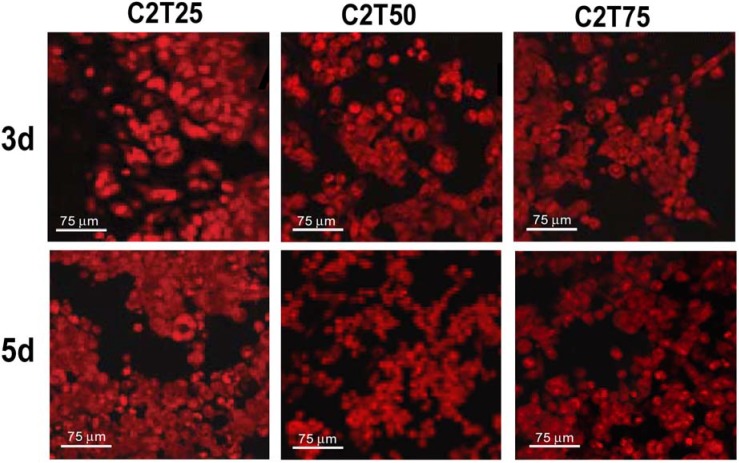
Confocal microscopy of J111 cells grown for 3 and 5 days on membranes made of different ratios of type I collagen from tendons and type II collagen from trachea cartilage (see Experimental Section). Cells were labeled with propidium iodide prior to microscopy observation.

**Figure 3:**
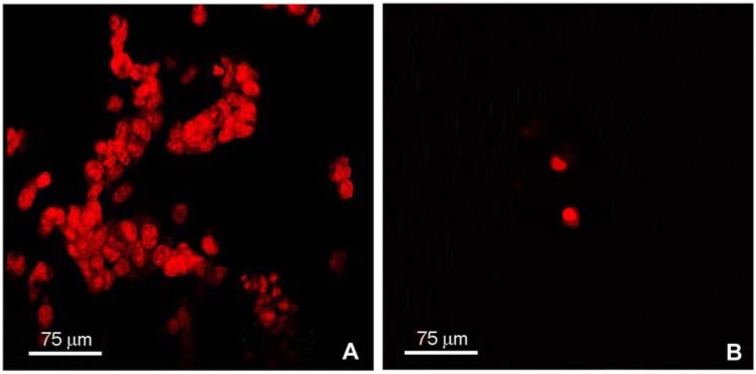
Confocal microscopy of J111 cells grown for 3 (A) and 5 (B) days on C2A25 membranes made of type I collagen from tendons and type II collagen from articular cartilage (see Experimental Section). Cells were labeled with propidium iodide prior to microscopy observation.

J111 cells, seeded on different substrates, were analysed by fluorescence (data not shown) as well as by confocal microscopy at days 3 and 5 of culture (Figure 1, Figure 2 and Figure 3). Data were very similar, independently from the morphological technique that was used, although images and resolution was obviously of better quality when samples were observed by confocal microscopy.

Cells grown on plastic surfaces exhibited good growth capabilities and adhesion on the substrate, and the number of cells progressively increased with time. At day 3 (Figure 1A), a consistent number of cells almost covered the surface. At day 5 (Figure 1B), the monolayer was completed; moreover, in some areas, cell aggregates could be observed.

J111 cells seeded on membranes made of collagens isolated from tendons and from trachea cartilage, and mixed at different ratios, always showed good viability and adhesion characteristics (Figure 2). Never the less, increased percentage of collagen from trachea cartilage was not associated to higher amounts of attached cells.

In particular, at day 5 the amount of cells was always higher compared to day 3, but the monolayer appeared better covered (approximately 85-90% of the whole surface) in the case of cells grown of C2T25 membranes made of 25% of type II collagen from trachea and of 75% type I collagen from tendons. When cells were grown on membranes with 50% (C2T50) and 75% (C2T75) of type II collagen from trachea, the covered surface, at day 5, reached 75-80% and 65-70%, respectively. Aggregates of cells were more frequently seen on C2T25 membranes, and only rarely on C2T50 scaffolds, whereas they were never observed on C2T75 membranes. From these data, membranes C2T25 appeared to be the most efficient for the growth of J111 cells.

By contrast, membranes made of collagens from tendons and articular cartilage appeared less suitable for the growth of J111 cells (Figure 3).

Already at day 3 (Figure 3A) the number of attached cells was significantly reduced compared to cells cultured on plastic or on membranes comprised of collagens from tendons and from trachea cartilage. At day 5 the number of cells present on membranes was dramatically reduced (Figure 3B), since the great majority of cells appeared detached from the substrate and floating in the medium. Similar results were obtained when collagens from tendons and from articular cartilage were mixed at different ratios (data not shown).

These observations suggested that membranes with the lower percentage of collagen from trachea cartilage (*i.e.,* C2T25 membranes) had the best properties for macrophage adhesion and proliferation. Therefore, in the second part of the present study, data obtained with membranes C2T25 will be reported.

### 2.2. Environmental Scanning Electron Microscopy (ESEM)

In order to investigate on the organization and distribution of J111 cells and on their morphology, observations by environmental scanning electron microscopy (ESEM) were performed after 1 and 3 days of culture. This technique allows to visualize at higher resolution, but reduced chemical and physical manipulations, cell morphology as well as interactions between cells and between cells and the substrate.

**Figure 4:**
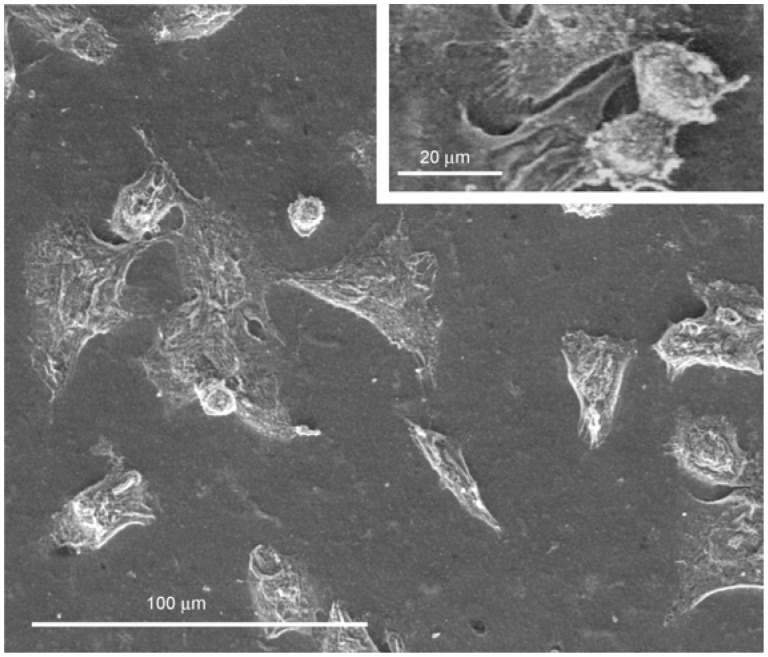
Environmental Scanning Electron Microscopy (ESEM) of J111 cells grown for 24 hours on collagen membranes C2T25.

After 24 hours from seeding, a discrete number of J111 cells could be observed on the surface of the collagen membrane C2T25. Of these cells, a few were round, whereas others had a triangular or polygonal shape, being flat and rather adherent to the substrate (Figure 4). Interestingly, the majority of cytoplasmic protrusion appeared to connect cells with the collagen membrane, whereas very few links were noted between cells (Figure 4 and 4 insert).

At day 3, the number of macrophages on the surface of the C2T25 membranes was markedly increased and cell distribution appeared more homogeneous in comparison to the previous time point.

**Figure 5:**
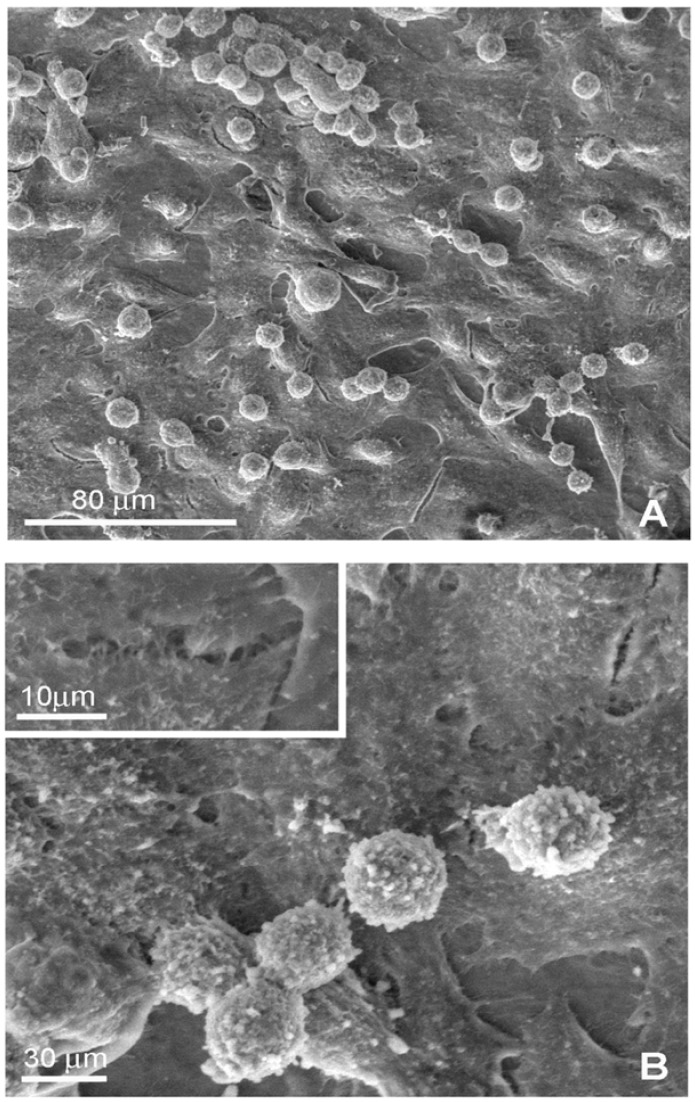
Environmental Scanning Electron Microscopy (ESEM) of J111 cells grown for 3 days on collagen membrane C2T25 at low (A) and high (B) magnification.

Moreover, the majority of cells showed a flat and polygonal shape and appeared in close contact one to the other forming an almost continuous monolayer (Figure 5A). At higher magnification, numerous cytoplasmic protrusions towards the substrate, and also between cells, were clearly visible (Figure 5B and 5B insert). These findings suggest a significant increase in cell-cell interactions and a good affinity of J111 cells towards this type of collagen membranes. As shown in Figure 5A, in some areas, there were round cells apparently on top of the flat monolayer. These macrophages are not detaching from the substrate, since they exhibit cytoplasmic expansions and protrusions connecting more cells and even forming cell aggregates (Figure 5A,B). It could be hypothesized that these are newly grown cells trying to find their place and to establish cell-cell and cell-matrix connections.

### 2.3. Macrophage proliferation

**Figure 6:**
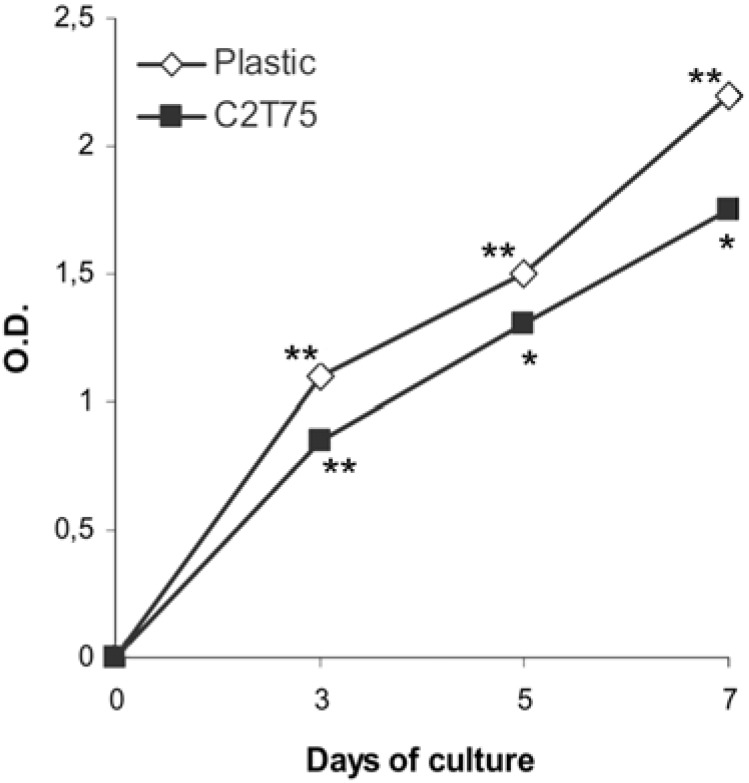
Proliferation of J111 cells grown up to seven days on plastic ( □ ) and on the collagen membrane C2T25 ( ■ ) evaluated by the MTT method. Data are expressed as mean values of four replicates. * p<0.05; ** p<0.01 compared to the previous time point.

The slope of the proliferation curves of J111 cells grown on plastic and of those grown on collagen membrane C2T25 was similar (Figure 6). However, proliferation of cells seeded on collagen was lower compared to cells cultured on plastic.

### 2.4. Cytokine production

**Figure 7:**
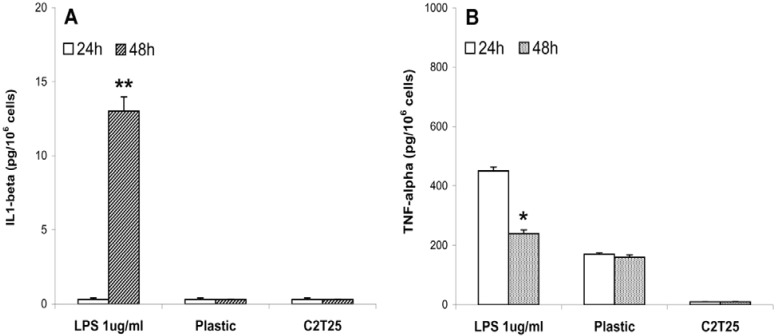
IL1-beta (A) and TNF-alfa (B) production by J111 cells grown for 24 and 48 hours on plastic and on the collagen membrane C2T25. Response to LPS was taken as a positive control. Data are expressed as mean values ± SD of four replicates. *p<0.05; **p<0.01 48 vs 24 hours.

The amounts of IL1-beta (Figure 7A) and TNF-alfa (Figure 7B) secreted by J111 cells, being related to cell activation and reactivity, have been measured after seeding of the cells on the collagen membrane C2T25 or on a plastic substrate. As a positive control cells have been exposed to LPS (1 μg/mL) for the whole length of the experiment, *i.e.,* 24 and 48 hours. Cells respond to LPS exposure by secreting significant amounts of both cytokines, although with a different time course. Production of TNF-alpha, in fact, seems to precede the synthesis of IL1-beta, which was detectable only after 48 hours. Moreover, TNF-alpha was secreted at higher level after 24 hours from LPS exposure, whereas after 48 hours values started to decrease. For cells grown on the plastic surface, at all time points, the amount of IL-1 beta was negligible, whereas a small amount of TNF-alpha was measured. Interestingly, production of both cytokines was never detected in J111 cells grown for 24 and 48 hours on the collagen membrane C2T25. These findings indicate that collagen membranes made of collagens typo I and II isolated from horse tendon and trachea, respectively, do not activate any response from J111 cells, further sustaining the good biocompatibility of these scaffolds.

Collagen is a biocompatible and bioactive non specific polymer that, either alone or in combination with other materials, occupies a foremost position in the field of tissue engineering. The use of collagen based biomaterials has been extensively reported in the literature, as a valuable aid in wound healing and in reconstructive surgery [20,21]. These materials, in form of three-dimensional matrix structures, have been used, for instance, as temporary scaffolds in order to improve tissue organization, as well as the function of damaged tissues or to favor new tissue formation. Never the less, development of these collagen-based biomaterials still requires a better understanding of the relationships between cells and substrates and between cells themselves, in the presence of a matrix scaffold. Collagen substrates, in fact, have been shown to influence proliferation, migration and differentiation of a number of different cells *in vitro* [22].

In the present investigation, we have studied the behavior of human macrophages of the cell line J111, after seeding on collagen membranes made of collagen type I and type II isolated from various sources. Collagen extracted from the trachea or from the articular cartilage is characterized by a different amount of crosslinks, values being higher in the trachea due to its structural function. Even though all cells, during the first days of culture, started to adhere to collagen membranes, the composition of these scaffolds influenced the number of attached cells, probably interfering with their affinity to the matrix. These findings became even more dramatic after 5 days of culture, when membranes comprised of collagen from articular cartilage were almost devoid of cells, whereas the percentage of collagen isolated from trachea cartilage was inversely proportional to the amount of attached cells. In the light of these results it could be suggested that, not only the type of collagen can exert a great influence of cell behaviour, but also the source and/or the tissue from which the matrix substrate is obtained. Interestingly, the proliferation rate of J111 cells on plastic or on collagen matrix was rather comparable, although cells on the matrix substrate proliferate slightly less. These observations are in accordance with data demonstrating that proliferation of fibroblasts cultured into collagen matrices is lower than that of cells grown in mono-layer on plastic [6,23], consistently with the hypothesis that the behaviour of cells within three-dimensional scaffolds, or even on the surface of matrix substrates, may be a more realistic estimate of the *in vivo* conditions compared to the traditional cell monolayer on plastic.

## 3. Experimental Section

### 3.1. Preparation of collagen membranes

Type I and II collagens were prepared by Opocrin SpA (Corlo, Modena, Italy). Briefly, insoluble type I collagen was isolated from horse Achilles’ tendon using neutral salt and diluted acid extractions, as previously described [24,25,26]. This tendon consists of about 87% collagen (evaluated on dry weight): 95% is highly cross-linked type I collagen, whereas less than 5% is type III coll [2]. Type II collagen was isolated from horse articular and trachea cartilage, in order to avoid problems related to materials of bovine origin. After chemical, physical and microbiological controls, type I and type II collagen gels were stratified in suitable templates, inserted into an electric field and maintained at a constant controlled temperature till membrane formation. These scaffolds were packed in blisters and sterilized with gamma rays. The following collagenous membranes were investigated: C2T25 (composed of 25% type II collagen from trachea and 75% type I collagen from tendons); C2T50 (composed of 50% type II collagen from trachea and 50% type I collagen from tendons); C2T75 (composed of 75% type II collagen from trachea and 25% type I collagen from tendons); C2A25 (composed of 25% type II collagen from articular tissue and 75% type I collagen from tendons); C2A50 (composed of 50% type II collagen from articular tissue and 50% type I collagen from tendons); C2A75 (composed of 75% type II collagen from articular tissue and 25% type I collagen from tendons).

The thickness of all membranes was 0.12-0.15 ± 0.03 mm. In addition, membranes were always characterized by a compact array of collagen fibrils, allowing cells to remain on the surface of the scaffold. Prior to use, collagen membranes, under sterile conditions, were washed twice in distilled water for 30 minutes and soaked overnight. Afterwards, the pH of water was controlled. Finally, films were washed with PBS three times for 15 minutes and, after the last washing, they were conditioned in growth culture medium, cut in small pieces of 18 × 20 mm and inserted into wells of appropriate size.

### 3.2. Macrophage culture

The J111 human monocyte/macrophage leukemia cell line was purchased at Centro Substrati Cellulari, Istituto Zooprofilattico Sperimentale, Brescia (Italy). Cells were cultured as mono-layer in Eagle’s Minimum Essential Medium (MEM) supplemented with 10% fetal bovine serum (FBS, Gibco, Grand Island, NY USA), 50 UI/mL penicillin, 50 μg/mL streptomycin, 2mM L-glutamine. Flasks of 75 cm^2^ (Becton Dickinson, Milano, Italy) were incubated at 37°C in humidified atmosphere (95% air and 5% CO_2_). Cells were routinely removed with Na-EDTA trypsin (0.05%) (Gibco) and split 1:2 weekly. In the experiments, 250.000 J111 cells were plated in “2 well-chamber slides” (Lab-Tek, Becton Dickinson) in a total volume of 2 mL of MEM with 10% FBS and antibiotics. In parallel, J111 cells were seeded on conditioned collagen membranes, in a total volume of 2 mL of MEM with 10% FCS and antibiotics. In both experimental conditions, the culture medium was renewed every day.

### 3.3. Fluorescence optical and confocal microscopy

At days 3 and 5, J111 cells seeded on collagen membranes were fixed with 3% paraformaldehyde for 30 minutes at room temperature, washed several times with PBS and, permeabilized with 1% Triton X-100 in PBS (Sigma Chemical, St Louis, MO, USA) for 30 minutes. After several washes with PBS, collagen- plated J111 cells were stained with 50 µg/mL propidium iodide (PI) (Sigma Chemical) for 2 hours and 30 minutes. PI binds to DNA producing an intense fluorescent signal, by contrast when the dye is unbound in an aqueous solution, it shows only a weak fluorescence. Membranes were observed with a fluorescence optical microscope (Leica DEMIL, Wetzlar Gmbh, Germany) and with a confocal microscope (Leica SP2, Wetzlar Gmbh Germany) [27,28].

### 3.4. Environmental Scanning Electron Microscopy (ESEM)

Cells grown on collagen membranes were fixed with 4% paraformaldehyde and 0.5% glutaraldehyde in Tyrode’s physiological solution, pH 7.3 for 30 min at room temperature.After washes in Tyrode’s solution, samples have been dehydrated in graded ethanol up to 70% ethanol in water and thereafter stained with uranyl acetate (saturated solution in 70% ethanol) for 90 min. Observations were made with a Quanta 200 ESEM (FEI Company, Eindhoven, Netherlands).

### 3.5. MTT assay

The tetrazolium-based colorimetric assay (MTT) is one of the methods of choice for the evaluation of cell survival and proliferation [29,30]. MTT (Sigma Chemical) is a yellow water-soluble tetrazolium dye which is reduced by live cells into a purple formazan product insoluble in aqueous solutions. The amount of generated formazan is directly proportional to the number of viable cells. At days 3, 5, and 7 of culture, the medium was removed and 2 mL of growth medium with 200 μL of MTT (5 mg/mL in PBS) were added to each sample. Cells were incubated, in the dark, at 37 °C in humidified atmosphere (95% air and 5% CO_2_) for six hours. Thereafter, the growth medium was removed and 2 mL of dimetyl sulphoxide (DMSO) (Sigma Chemical) were added to each well to dissolve purple crystals of formazan. Absorbance was measured in a spectrophotometer at 540 nm wavelength. Results were expressed as O.D. (optical density) after blank (collagen membrane only) subtraction. Reported values are the mean of 4 replicates. Significance of differences between time points was assessed by the Student’s t-test.

### 3.6. Cytokine production

J111 cells were plated at a density of 250 × 10^3^ cells on plastic surfaces and on collagen membranes in a total volume of 2 mL of MEM with 10% FCS and antibiotics. After 24 and 48 hours, media were collected and the level of IL1β and TNFα determined by ELISA according to manufacturer’s protocols (R&D Systems, Minneapolis, MN, USA). Reported values are the mean of 4 replicates. As a positive control, cells were grown in the presence of 1µg of lipopolysaccharide (LPS) (Sigma Chemical) added to the medium. Significance of differences was assessed by the Student’s t-test.

## 4. Conclusions

In conclusion, the model presented here represents a dynamic system to investigate the interactions and the reactivity of cells with biomaterials and to obtain qualitative and quantitative information regarding cell response as well as the biocompatibility of matrices that can be used as substrates for cell adhesion and proliferation.
